# Early identification of macrophage activation syndrome in adult-onset Still’s disease: a case report and literature review

**DOI:** 10.3389/fmed.2025.1498928

**Published:** 2025-01-27

**Authors:** Ting Long, Jing Xu, Bo-Zhi Lin, Sheng-Guang Li

**Affiliations:** ^1^Department of Rheumatology and Immunology, Peking University International Hospital, Beijing, China; ^2^Department of Clinical Laboratory, Peking University International Hospital, Beijing, China

**Keywords:** adult-onset Still’s disease (AOSD), macrophage activation syndrome (MAS), hemophagocytic lymphohistiocytosis (HLH), HScore, MS score, cytomegalovirus (CMV) infection

## Abstract

**Background:**

Adult-onset Still’s disease (AOSD) is a rare systemic inflammatory disorder characterized by high spiking fevers, evanescent rash, and polyarthritis. A serious complication of AOSD is macrophage activation syndrome (MAS), a life-threatening hyperinflammatory condition that can lead to multiorgan failure if not promptly diagnosed and treated.

**Objective:**

This case report and literature review focus on the early identification of MAS in patients with AOSD, highlighting diagnostic challenges, differential diagnoses, and the utility of scoring systems like the HScore and MS score in clinical practice.

**Case presentation:**

We report the case of an 80-year-old woman who presented with a complex diagnostic challenge involving AOSD complicated by MAS and concurrent cytomegalovirus (CMV) infection. Her clinical course was marked by recurrent high fevers, cytopenias, hyperferritinemia, and liver dysfunction. Despite extensive workup, initial diagnoses of infections and autoimmune conditions were considered and ruled out. The HScore and MS score were calculated to be 210 and 1.607, respectively, both indicative of MAS. The patient was treated according to the HLH-94 protocol, with high-dose dexamethasone and etoposide, alongside broad-spectrum antimicrobial and antiviral therapy. She responded well to treatment, with resolution of fever and improvement in clinical symptoms.

**Discussion:**

The overlap between AOSD and MAS symptoms complicates early diagnosis, making scoring systems critical in differentiating MAS from other conditions. The HScore and MS score provided a structured approach to diagnosis, guiding timely intervention and improving the patient’s prognosis. Our literature review emphasizes the importance of early recognition and integration of these scoring systems into routine clinical practice to enhance outcomes.

**Conclusion:**

This case underscores the necessity of early identification and intervention in MAS associated with AOSD. The application of diagnostic scoring systems like the HScore and MS score is essential for prompt diagnosis and effective treatment, ultimately improving patient survival rates.

## Introduction

Adult-onset Still’s disease (AOSD) is a rare systemic autoinflammatory disorder characterized by high spiking fevers, evanescent rash, arthralgia, and systemic inflammation. While AOSD can often be managed with immunosuppressive therapy, it is sometimes complicated by macrophage activation syndrome (MAS), a severe and potentially life-threatening hyperinflammatory condition (1). MAS, also known as secondary hemophagocytic lymphohistiocytosis (HLH), is marked by uncontrolled immune activation that can rapidly progress to multiorgan failure if not promptly diagnosed and treated (2).

Diagnosing MAS in the context of AOSD is particularly challenging due to overlapping clinical features between the two conditions (3). Symptoms such as persistent fever, cytopenias, hyperferritinemia, and liver dysfunction can be seen in both AOSD and MAS, complicating early diagnosis. Traditional diagnostic criteria for HLH, such as the HLH-2004 criteria, have proven to be insufficient for early recognition of MAS in adults (4). To address this limitation, alternative diagnostic tools like the HScore and MS score have been developed and validated to enhance early detection of MAS in adult patients with rheumatic conditions such as AOSD (5–7).

**Figure 1:**
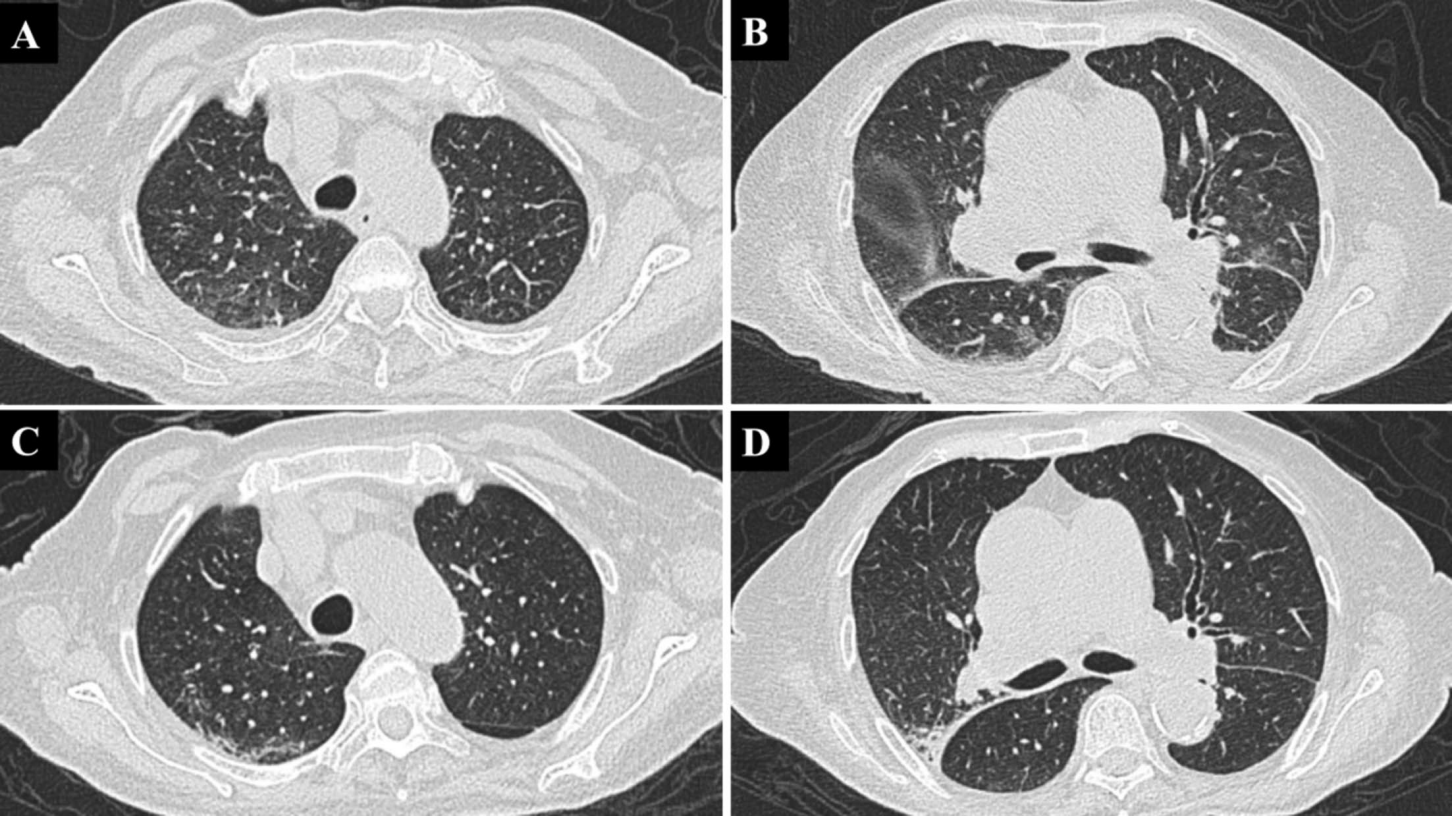
Chest CT of the patient. **(A,B)** These images were taken on the third day of admission and show diffuse ground-glass opacities throughout both lungs, accompanied by small pleural effusions bilaterally. **(C,D)** The follow-up chest CT images, taken during the second week of admission, demonstrate a significant reduction in the diffuse infiltrates and complete resolution of the pleural effusions seen earlier.

**Figure 2 fig2:**
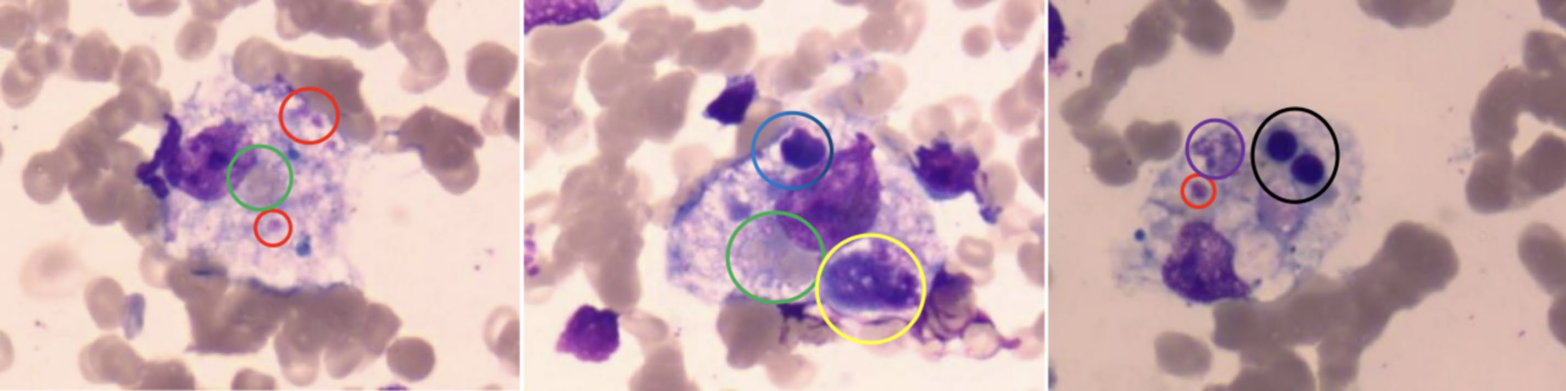
Bone marrow biopsy showing hemophagocytosis. This image represents a bone marrow biopsy stained with Rich’s-Giemsa at 1000× magnification, highlighting the characteristic hemophagocytosis observed in hemophagocytic syndrome. Several histiocytes are seen engulfing different cell types, indicated by colored circles: Red circles: phagocytosed platelets. Green circles: mature erythrocytes. Yellow circles: immature granulocytes. Blue circles: mature lymphocytes. Black circles: late erythroblasts. Purple circles: degenerated cells.

**Figure 3:**
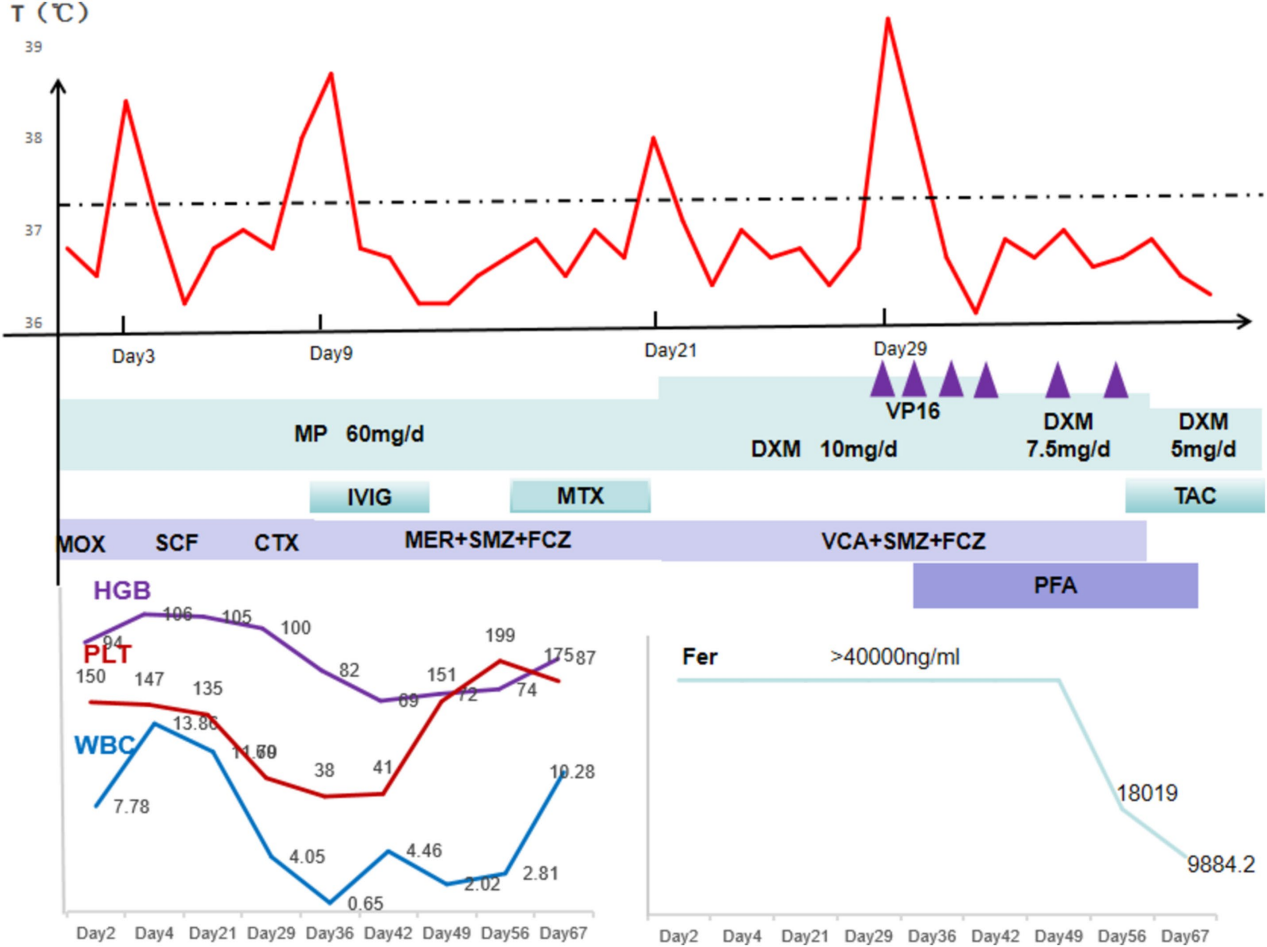
Schematic diagram of the trend of changes in treatment, body temperature, and laboratory test results. MP, methylprednisolone; DXM, dexamethasone; MOX, moxifloxacin; SC, Cefperazone-Sulbactam; CTX, cefotaxime; MER, meropenem; SMZ, trimethoprim-sulfamethoxazole; FCZ, fluconazole; VCA, vancomycin; MTX, methotrexate; PFA, phosphonoformic acid; TAC, tacrolimus.

The importance of early diagnosis and intervention in MAS cannot be overstated, as delayed treatment significantly worsens prognosis. The HScore and MS score provide clinicians with structured approaches to assess the likelihood of MAS based on clinical and laboratory findings, including hyperferritinemia, cytopenias, and organ involvement. Recent studies have demonstrated the effectiveness of these scoring systems in guiding timely treatment, reducing mortality, and improving outcomes (7, 8). However, the utility of these tools in distinguishing MAS from other conditions with overlapping features, such as severe infections or malignancies, remains a critical area of discussion (Figures 1 [Fig fig2]–3).

This paper presents the case of an 80-year-old woman with AOSD complicated by MAS and concurrent cytomegalovirus (CMV) infection. The case underscores the diagnostic challenges posed by overlapping clinical features, the utility and limitations of scoring systems such as the HScore and MS score, and the importance of prompt therapeutic intervention. By integrating a comprehensive literature review with insights from this case, we aim to highlight the need for further validation and refinement of diagnostic tools for MAS in AOSD patients, as well as explore emerging therapeutic strategies tailored to this complex condition.

## Case presentation

An 80-year-old woman presented with a one-year history of intermittent fever, worsening over the past 20 days. Initially, in October 2022, after cold exposure, she experienced fevers up to 38.9°C, accompanied by a persistent cough, muscle and joint pain, and a generalized pruritic erythematous rash. Laboratory tests at that time revealed leukocytosis (white blood cell count [WBC] 11.7 × 10^9/L, neutrophil count 10.03 × 10^9/L), elevated C-reactive protein (CRP 113.9 mg/L), and lactate dehydrogenase (LDH 523 U/L). Renal function and infection markers were normal. Treatment with antibiotics and corticosteroids led to symptom resolution.

Less than a month later, fever recurred (38.0–38.8°C). Laboratory tests showed elevated CRP (18.8 mg/L), erythrocyte sedimentation rate (ESR 72 mm/h), and serum ferritin (3,793.9 ng/mL). A PET-CT scan revealed enlarged mediastinal and hilar lymph nodes with increased uptake, suggestive of chronic inflammation. Celecoxib treatment provided minimal improvement.

In January 2023, she was admitted with high fever (39.6°C) and altered consciousness. Laboratory findings included WBC 15.17 × 10^9/L, neutrophil count 14.64 × 10^9/L, ferritin 23,712 ng/mL, ESR 110 mm/h, CRP 163.3 mg/L, and D-dimer 6,153 ng/mL. Bone marrow examination occasionally revealed hemophagocytic cells, which supported but was not required for the diagnosis of MAS. Despite antibiotic and supportive therapy, fever persisted intermittently after discharge.

By July 2023, an erythematous rash had developed on her trunk. Immunological assays showed significant reductions in B and T cells, with elevated IgG (27.55 g/L). Intravenous immunoglobulin (IVIG) temporarily resolved the fever. However, by January 2024, the fever had worsened, peaking at 39.6°C, with symptoms including drowsiness, polyarthralgia, and erythematous rashes. Laboratory tests showed hemoglobin 104 g/L, platelet count 147 × 10^9/L, CRP 148.3 mg/L, and ferritin >40,000 ng/mL. Methylprednisolone treatment provided temporary relief.

Initially, MAS-specific diagnostic tools like HScore and MS Score were not used to evaluate the patient’s condition. Instead, the diagnostic process followed established MAS criteria. Besides, the diagnosis of AOSD remained uncertain due to ongoing concerns about possible infections or malignancies. A definitive diagnosis was made only after the appearance of new erythematous rashes, arthritis, progressive reduction of blood cells and fibrinogen, and the detection of hemophagocytic cells in the bone marrow.

During her hospital stay, serial laboratory tests revealed dynamic changes (Table 1). On admission (Day 2), the HScore was retrospectively calculated at 210 (Table 2), primarily driven by fever, ferritin levels, and bone marrow findings. By Day 29, the HScore had risen to 253 due to worsening cytopenias and hypoalbuminemia, prompting initiation of the HLH-94 protocol (high-dose dexamethasone and etoposide). MS Score dynamics further supported the diagnosis (Table 3).

**Table 1:** The main test results at different stages during the patient’s hospitalization.

Variable	Reference range	Day 2	Day 4	Day 21	Day 29	Day36 (1 week after HLH94 regiment)	Day 42 (2 weeks after HLH94 regiment)	Day 49 (3 weeks after HLH94 regiment)	Day56 (4 weeks after HLH94 regiment)	Day 67 (the day before discharge)	6 months after discharge
White-cell count (×10^9/L)	3.50–9.50	7.78	13.86	11.79	4.05	0.65	4.46	2.02	2.81	10.28	8.05
Neutrophil count (10^9/L)	3.50–9.50	6.60	12.87	11.45	3.50	0.42	3.36	1.45	2.10	8.03	3.73
Hemoglobin (g/L)	115–150	94.00	106.00	105.00	100.00	82.00	69.00	72.00	74.00	87.00	101
Platelet count (10^9/L)	125–350	150.00	147.00	135.00	60.00	38.00	41.00	151.00	199.00	175.00	135
Protein (g/L)											
Total	65–85	71.10	78.50	67.80	54.00	64.80	53.40	55.40	57.30	60.10	65.5
Albumin	40–55	31.50	34.50	32.30	26.50	30.60	28.60	30.60	31.20	33.90	38.8
Alanine aminotransferase (U/liter)	7–40	19.00	18.00	28.00	18.00	18.00	16.00	20.00	15.00	36.00	39
Aspartate aminotransferase (U/liter)	13–35	24.00	35.00	39.00	66.00	34.00	40.00	26.00	21.00	34.00	20
Alkaline Phosphatase (U/liter)	50–135	82.00	87.00	91.00	76.00	78.00	81.00	87.00	76.00	105.00	83
Glutamyl Transferase (U/liter)	7–45	48.00	49.00	70.00	54.00	53.00	41.00	84.00	79.00	120.00	107
Bilirubin (μmol/L)											
Total	3.4–23.3	5.50	7.50	6.70	5.70	7.90	7.60	4.90	7.20	6.20	8.4
Direct	0.0–6.8	2.10	2.80	2.00	1.90	2.70	2.70	1.90	2.30	1.90	2.7
Lactate dehydrogenase (U/liter)	120–250	313.00	/	365.00	739.00	429.00	771.00	457.00	441.00	355.00	310
C-reactive protein (mg/liter)	≤10.00	26.05	50.62	11.04	38.26	10.79	40.52	11.50	9.06	2.92	0.78
Erythrocyte sedimentation rate (mm/h)	0–20	62.00	/	/	/	/	/	/	/	33.00	/
Serum Ferritin (ng/mL)	21.8–274.7	>40,000	/	>40,000	/	>40,000	>40,000	>40,000	18019.00	9884.20	773.3
NK Cell Activity (pg/mL)	25.65–313.37	80.00	/	/	/	/	/	/	/	/	/
sCD25 (%)	≥15.11	6.32	/	/	/	/	/	/	/	/	/
Triglycerides (mmol/L)	<1.7	1.62	1.32	3.78	2.86	3.06	2.74	2.37	2.17	2.13	2.23
Fibrinogen (mg/dL)	200–400	239.00	252.00	194.00	162.00	123.00	282.00	236.00	/	315.00	315
D-dimer (ng/mL)	<250	2229.00	2986.00	738.00	3045.00	662.00	2646.00	666.00	/	280.00	161
Procalcitonin (ng/mL)	≤0.05	0.07	0.19	0.24	0.30	0.22	0.08	/	/	0.03	/
CMV-DNA (copies/mL)	<4E+02	<4E+02	/	<4E+02	1.78E +4	/	/	1.46E +2	7.4E +1	<4E+02	/

**Table 2:** HScore of the patients at admission.

Parameter	No. of points (criteria for scoring)	Scores of Day 2	Scores of Day 29	Scores of Day 67
Known underlying immunosuppression*	0 (no) or 18 (yes)	18	18	18
Temperature (°C)	0 (<38.4), 33 (38.4–39.4), or 49(>39.4)	33	33	0
Organomegaly	0(no),23(hepatomegaly or splenomegaly), or 38(hepatomegaly and splenomegaly)	0	0	0
No. of cytopeniast	0 (1 lineage), 24 (2 lineages), or 34 (3 lineages)	0	24	0
Ferritin (μg/L)	0(<2,000), 35 (2,000–6,000), or 50(>6,000)	50	50	50
Triglyceride (mmol/L)	0 (<1.5), 44 (1.5–4), or 64 (>4)	44	44	44
Fibrinogen (g/L)	0 (>2.5) or 30 (≤2.5)	30	30	0
Aspartate aminotransferase (U/L)	0 (<30) or 19 (≥30)	0	19	19
Hemophagocytosis on bone	0 (no) or 35 (yes) marrow aspirate	35	35	Not performed
Hscore		210	253	131

**Table 3:** MS score of the patients at admission.

		Day2		Day29		Day67	
Parameter	Weight coefficient	Score	Weighted scores	Score	Weighted scores	Score	Weighted scores
CNS involvement	2.44	0	0	1	2.44	0	0
Hemorrhagic manifestations	1.54	0	0	0	0	0	0
Arthritis	−1.3	1	−1.3	0	0	0	0
PLT count	−0.003	150	−0.45	60	−0.18	175	−0.525
LDH	0.001	313	0.313	739	0.739	355	0.355
Fibrinogen	−0.004	239	−0.956	162	−0.648	315	−1.24
Ferritin	0.0001	40,000	4	40,000	4	9884.2	0.98842
MS score		1.607		6.351		−0.44158	

Additional complications arose, including carbapenem-resistant *Klebsiella pneumoniae* and methicillin-resistant *Staphylococcus aureus* infections, managed with targeted antimicrobial therapy. Following HLH-94 treatment, significant clinical improvement was observed, including normalization of CRP, ESR, and ferritin levels (Table 1).

The retrospective analysis of this case revealed that treatment delays occurred due to diagnostic uncertainties and the lack of early application of scoring systems like HScore and MS Score. This experience highlighted the need to improve diagnostic approaches for MAS in the context of AOSD, enabling earlier recognition and intervention.

By Day 67, the patient’s HScore had decreased to 131, and she was discharged on low-dose dexamethasone and tacrolimus. At a six-month follow-up, she remained asymptomatic, with normalized laboratory parameters, including ferritin (773.3 ng/mL).

This case underscores the importance of timely diagnosis and dynamic monitoring using HScore and MS Score. It highlights the need for integrated therapeutic strategies tailored to evolving clinical findings, ensuring improved patient outcomes in complex cases of AOSD-associated MAS.

## Discussion

The diagnosis and management of macrophage activation syndrome (MAS) in the context of adult-onset Still’s disease (AOSD) remain significant clinical challenges. MAS is a hyperinflammatory syndrome characterized by clinical and laboratory features that overlap with those of AOSD, such as persistent fever, hyperferritinemia, and cytopenias (9). This overlap often complicates timely diagnosis and treatment.

In this case, delays in MAS diagnosis were attributed to the reliance on traditional MAS diagnostic criteria, while newer tools such as HScore and MS Score (8, 10) were not applied during the initial evaluation. Retrospective analysis of this case highlighted the utility of these scoring systems in providing an objective framework for early MAS identification, even before indicators supporting diagnosis become obvious, such as hemophagocytosis in bone marrow aspirate (11, 12). Studies have shown that HScore and MS Score offer high sensitivity and specificity for diagnosing MAS in adult patients with rheumatologic conditions, making them valuable tools in clinical practice (13–15).

## Balancing the use of HScore and MS score

While HScore and MS Score are valuable diagnostic tools, their limitations must be understood to maximize their utility (16). One key limitation is their inability to fully distinguish MAS from other conditions with overlapping features, such as severe infections or hematologic malignancies. These overlaps can lead to diagnostic ambiguity, especially in cases with elevated ferritin and inflammatory markers, which are common in both MAS and other systemic illnesses.

Additionally, these scores rely on specific thresholds for laboratory and clinical findings, which might not reflect the dynamic progression of MAS. For example, in this case, the HScore on Day 2 indicated a high likelihood of MAS, but the lack of confirmatory findings delayed treatment initiation. Dynamic use of these scores, with repeated assessments over time, could improve diagnostic accuracy and guide timely interventions.

Another consideration is the need for further validation of these tools in AOSD-associated MAS. While both HScore and MS Score were developed for broader applications, their sensitivity and specificity in AOSD remain underexplored. Integrating additional biomarkers, such as Interleukin-18, Pentraxin-3, CXCL9, CD38 + HLA-DR+ cells, and adenosine deaminase 2 activity, which have been studied as potential biomarkers for distinguishing AOSD with MA from other forms of HLH caused by different etiologies (17–22).

## Improving early diagnosis and monitoring

Future efforts should focus on integrating HScore and MS Score into routine clinical practice while addressing their current limitations. Machine learning models that incorporate dynamic changes in laboratory and clinical data could help create personalized diagnostic algorithms. Such advancements could reduce diagnostic uncertainty and ensure earlier treatment.

## Therapeutic implications

Therapeutically, the HLH-94 protocol remains a cornerstone of MAS treatment, involving high-dose glucocorticoids, etoposide, and cyclosporine (13). In this case, high-dose dexamethasone and etoposide were selected due to the patient’s severe disease and organ involvement. Emerging therapies, such as IL-1 inhibitors (anakinra) IL-6 inhibitors (tocilizumab), anti-IFN-*γ* neutralizing monoclonal antibodies (Emapalumab), and JAK1/JAK2 inhibitors (Ruxolitinib) have shown promise in treating MAS associated with AOSD or other conditions (23–26). However, their use in this case was limited by unavailability or concerns about potential infection risks.

Dynamic monitoring of MAS markers, including ferritin, fibrinogen, and pentraxin 3 played a critical role in assessing treatment response and guiding therapeutic adjustments (19). This case demonstrated that HScore and MS Score are not only diagnostic tools but also valuable for monitoring disease progression and therapeutic efficacy.

## Conclusion

This case underscores the importance of integrating advanced diagnostic tools, such as HScore and MS Score, into the management of AOSD-associated MAS. While these tools have limitations, their judicious use can facilitate earlier diagnosis and improve patient outcomes. Future research should focus on refining these tools, exploring novel biomarkers, and comparing therapeutic strategies to enhance care for patients with complex syndromes like AOSD-associated MAS.

## Data Availability

The datasets presented in this study can be found in online repositories. The names of the repository/repositories and accession number(s) can be found in the article/supplementary material.
