# Corrigendum: Early identification of macrophage activation syndrome in adult-onset Still's disease: a case report and literature review

**DOI:** 10.3389/fmed.2025.1600683

**Published:** 2025-04-09

**Authors:** Ting Long, Jing Xu, Bo-Zhi Lin, Sheng-Guang Li

**Affiliations:** ^1^Department of Rheumatology and Immunology, Peking University International Hospital, Beijing, China; ^2^Department of Clinical Laboratory, Peking University International Hospital, Beijing, China

**Keywords:** adult-onset Still's disease (AOSD), macrophage activation syndrome (MAS), hemophagocytic lymphohistiocytosis (HLH), HScore, MS score, cytomegalovirus (CMV) infection

In the published article, there was an error in affiliation(s) 1 and 2. Instead of “1 Department of Rheumatology and Immunology, International Hospital, Peking University, Beijing, China, 2 Department of Clinical Laboratory, International Hospital, Peking University, Beijing, China”, it should be “1 Department of Rheumatology and Immunology, Peking University International Hospital, Beijing, China, 2 Department of Clinical Laboratory, Peking University International Hospital, Beijing, China”.

The authors apologize for this error and state that this does not change the scientific conclusions of the article in any way. The original article has been updated.

